# Antithrombotic strategies and outcomes in neonates and infants with cardiac shunts: a systematic review and meta-analysis

**DOI:** 10.1016/j.rpth.2025.103161

**Published:** 2025-08-26

**Authors:** Amy L. Kiskaddon, Neil A. Goldenberg, Marisol Betensky, Joshua W. Branstetter, Dina Ashour, Pamela Williams, Arabela C. Stock, Michael Silvey, Therese M. Giglia, Nhue L. Do, Cardiac Disease Thrombosis and Hemostasis Working Party of the ISTH SSC Subcommittee on Pediatric and Neonatal Thrombosis and Hemostasis, Dalia Bashir, Kisha Beg, Victor Benvenuto, Marisol Betensky, Rukhmi Bhat, Joshua Branstetter, Paul Davies, Nhue Do, Kate Garland, Therese Giglia, Neil Goldenberg, Amy Kiskaddon, Joel Livingston, Christoph Male, Paul Monagle, Michael Silvey, Arabela Stock, Jun Teruya, Gary Woods

**Affiliations:** 1 Divisions of Hematology and Cardiology, Department of Pediatrics, Johns Hopkins University School of Medicine, Baltimore, Maryland, USA; 2 Institute for Clinical and Translational Research, Johns Hopkins All Children’s Hospital, St. Petersburg, Florida, USA; 3 Department of Pharmacy, Johns Hopkins All Children’s Hospital, St. Petersburg, Florida, USA; 4 Heart Institute, Johns Hopkins All Children’s Hospital, St. Petersburg, Florida, USA; 5 Division of Hematology, Departments of Pediatrics and Medicine, Johns Hopkins University School of Medicine, Baltimore, Maryland, USA; 6 Cancer and Blood Disorders Institute, Johns Hopkins All Children’s Hospital, St. Petersburg, Florida, USA; 7 Division of Hematology, Departments of Pediatrics, Johns Hopkins University School of Medicine, Baltimore, Maryland, USA; 8 Department of Pharmacy, Children’s Healthcare of Atlanta, Atlanta, Georgia, USA; 9 Institute for Clinical and Translational Research, Epidemiology and Biostatistics Shared Resource, Johns Hopkins All Children’s Hospital, St. Petersburg, Florida, USA; 10 Medical Library, Johns Hopkins All Children’s Hospital, St. Petersburg, Florida, USA; 11 Division of Cardiac Critical Care, Johns Hopkins All Children’s Hospital, St. Petersburg, Florida, USA; 12 Division of Hematology/Oncology/Bone Marrow Transplant, Department of Pediatrics, Children’s Mercy Hospital, Kansas City, Missouri, USA; 13 Division of Cardiology, Department of Pediatrics, The Children’s Hospital of Philadelphia, Perelman School of Medicine at the University of Pennsylvania, Philadelphia, Pennsylvania, USA; 14 Advocate Children’s Heart Institute, Advocate Children’s Hospital, Chicago, Illinois, USA; 15 Chicagoland Children’s Health Alliance, Chicago, Illinois, USA

**Keywords:** cardiac shunt, congenital heart disease, infant, neonate, shunt thrombosis, thromboprophylaxis

## Abstract

**Background:**

Cardiac shunt thrombosis in neonates and infants remains a concern for shunt failure and mortality. The optimal strategy for thromboprophylaxis remains unknown.

**Objectives:**

This systematic review aims to characterize antithrombotic strategies and outcomes in neonates and infants with a cardiac shunt.

**Methods:**

MEDLINE, Embase, and Cochrane CENTRAL were searched from inception through July 2024 for studies reporting shunt thrombosis prevalence among infants who received a cardiac shunt. We estimated the pooled prevalence of shunt thrombosis using random-effects meta-analysis. In the subgroup analysis, we evaluated the effects of shunt type and antithrombotic strategies on shunt thrombosis prevalence.

**Results:**

A total of 39 studies (29 retrospective, 10 prospective) were included, totaling 4735 patients. The most common shunt type was the modified Blalock-Taussig (*n* = 2224, 47%). Mortality related to shunt thrombosis occurred in 102 (26.2%) patients with shunt thrombosis. The most common antithrombotic agents in the acute postoperative setting were unfractionated heparin (UFH; *n* = 1452, 30.7%) and aspirin (*n* = 1413, 29.3%). The pooled prevalence of shunt thrombosis was 8.4% (95% CI, 6.5%-10.4%) and varied among antithrombotic agents: aspirin 7.4% (95% CI, 4.0%-11.4%), UFH 3.8% (95% CI, 0%-12.3%), or UFH followed by aspirin 6.3% (95% CI, 3.6%-9.4%).

**Conclusions:**

This systematic review of nearly 5000 neonates and infants reveals a high rate of mortality associated with shunt thrombosis. Collaborative prospective studies are warranted to evaluate antithrombotic regimen–outcome relationships and prognostic factors for shunt thrombosis and bleeding outcomes in these children.

## Essentials

• Pediatric cardiac shunt thrombosis poses a risk of death; optimal prevention strategies unknown.
• This review of nearly 5000 neonates and infants shows high mortality from shunt thrombosis.
• Thirty nine studies showed shunt thrombosis occurred in 8.4% with 26.2% mortality: aspirin 7.4%, unfractionated heparin 3.8%.
• Prospective studies are needed to assess antithrombotic strategies and prognostic factors.

## 1 Introduction

Children with congenital heart disease (CHD) have an increased thrombosis risk due to altered blood flow, a hypercoagulable state, surgery, foreign materials, and central lines. Among patients with CHD, neonates and infants with complex cyanotic CHD have high rates of thrombosis and mortality [1]. Neonates and infants with complex cyanotic CHD who are not suitable for a complete repair often undergo palliative intervention to connect the systemic artery or ventricle to the pulmonary arteries to establish stable pulmonary blood flow [[2], [3], [4], [5], [6], [7], [8]]. Common surgeries to supply stable systemic-to-pulmonary artery blood flow include aortopulmonary shunts, ductus arteriosus stents, and right ventricle-to-pulmonary artery conduits [3]. Shunt thrombosis continues to be a major complication after palliative congenital heart surgery and remains an incompletely understood phenomenon accounting for significant morbidity and mortality [4]. The rate of thromboembolic events in systemic-to-pulmonary artery shunts is reported to be as high as 50% [[4], [5], [6], [7], [8]]. Morbidities associated with thrombosis in the single ventricle CHD population include pulmonary embolism, stroke, vascular inaccessibility, and increased resource requirements [9,10]. Shunt thrombosis is also known to be the cause of mortality in this population, with rates ranging from 21% to 40% [7,11].

Thromboprophylaxis for the prevention of shunt thrombosis is not an accepted standard due to a lack of high-level evidence. A landmark study demonstrated that aspirin, compared with no thromboprophylaxis, reduced the risk of shunt thrombosis 7-fold [12]. Despite the use of various antiplatelet and anticoagulant agents, thrombosis remains a common complication. In addition, evidence is limited on the ideal antithrombotic approach for cardiac shunts, and there is variation in the agent selection and dosing utilized. Ultimately, the optimal strategy for thromboprophylaxis remains unknown.

This review aimed to characterize types of antithrombotic agents and dosing as well as outcomes in neonates and infants with CHD and a cardiac shunt.

## 2 Methods

### 2.1 Search strategies

The search strategies were designed and performed by a medical librarian trained in searching electronic databases for systematic reviews. MEDLINE (OVID interface) was searched from inception through December 18, 2023. A predetermined list of antithrombotic drugs and specific types of cardiac shunts was developed by the team prior to the literature search. The MEDLINE strategy included a combination of medical subject headings and keywords to capture the concepts of surgical shunts and anticoagulant agents in the neonatal and infant population. Articles were limited to English and French languages or foreign articles with English abstracts. The MEDLINE search was adapted for Embase (Elsevier) and CENTRAL (Cochrane Central Register of Controlled Trials). All searches were updated on July 31, 2024. The complete database search strategies are presented in the supplementary material. The guideline recommended by the Preferred Reporting Items for Systematic Reviews and Meta-Analyses was utilized [13]. The references of the included articles were reviewed to identify additional publications of relevance.

### 2.2 Inclusion and exclusion criteria

Full-text articles were eligible for inclusion if they were retrospective or prospective and included: (a) patients <12 months of age with a congenital heart defect who received a cardiac shunt, (b) shunt type and size, (c) antithrombotic agent, dosing, and laboratory monitoring strategy, and (d) bleeding, shunt thrombosis, mortality, and shunt thrombosis-related mortality. For this review, cardiac shunt was defined as an intervention to redirect blood to the lungs (either systemic to lungs or right ventricle to lungs). Articles were included if they reported patient-level data that met the inclusion criteria. Case reports, narrative reviews, conference abstracts, and commentaries were excluded.

### 2.3 Selection and screening of studies

Two reviewers (A.L.K. and N.L.D.) independently examined the titles and abstracts of all studies generated from the database searches to identify potentially relevant studies. The authors independently assessed the manuscripts for all potentially relevant studies. Any differences in opinion regarding studies to include were resolved by discussion and/or with a third reviewer (N.A.G). All studies were assessed for quality by 2 reviewers (A.L.K. and N.L.D.) using the National Institutes of Health Quality Assessment Tool for Observational and Cross-Sectional Studies.

### 2.4 Data extraction and management

Using a structured data form for this project, 2 authors (A.L.K. and N.L.D) independently extracted the following information from the articles: study design, study participant demographics (ie, age, weight), congenital heart defect, shunt type and size, antithrombotic agent and dose, antithrombotic monitoring, and clinical outcomes (ie, shunt thrombosis, mortality, mortality related to shunt thrombosis). Any differences were resolved by discussion and/or consensus with a third reviewer (N.A.G.) if necessary. The classification of bleeding and shunt thrombosis was extracted as reported or defined by the authors of each study.

### 2.5 Statistical analysis

We estimated the pooled prevalence with 95% CIs using the method of random effects to calculate the study weights. Heterogeneity was evaluated using *I*^2^ statistic and Cochrane Q chi-squared test for heterogeneity. We conducted subgroup analyses to estimate the pooled prevalence by antithrombotic strategy and shunt types, as well as to explore other causes of heterogeneity, including study quality, timing of shunt thrombosis (early vs late), age (neonates vs infants), and shunt size. Meta-analysis was conducted using MetaXL 5.3 (EpiGear International).

## 3 Results

### 3.1 Search results

**Figure 1:**
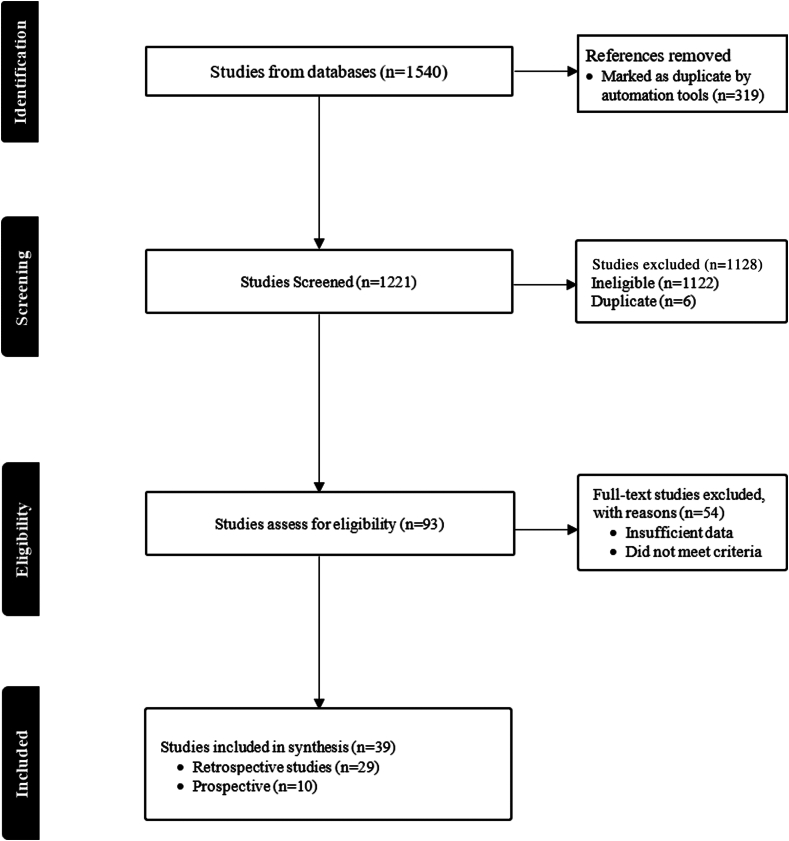
Prisma Flowchart.

The search strategy identified a total of 1540 publications. Of these, 319 were determined to be duplicates, leaving 1221 for screening. One thousand one-hundred twenty-eight were excluded based on the article type, the study population, and the information provided as defined by protocol inclusion and exclusion requirements. A total of 93 articles were deemed eligible for inclusion, among which 54 were excluded based on a lack of predetermined patient-level data of interest. The final review consisted of 39 publications, of which the majority were retrospective cohorts (*n* = 29), and the remainder were prospective studies (*n* = 10) (Figure 1) [2,7,[14], [15], [16], [17], [18], [19], [20], [21], [22], [23], [24], [25], [26], [27], [28], [29], [30], [31], [32], [33], [34], [35], [36], [37], [38], [39], [40], [41], [42], [43], [44], [45], [46], [47], [48], [49]]. There were 2684 (56.7%) and 2051 (43.3%) patients from the retrospective and prospective studies, respectively. All articles were published between the years 1978 and 2023.

### 3.2 Assessment of quality

Individual study quality was assessed using the National Institutes of Health Quality Assessment Tool for Observational Cohort and Cross-Sectional Studies. Of the 39 studies in this systematic review, 12 (30.8%) were graded as good (Supplementary Table 1).

### 3.3 Patient and antithrombotic treatment characteristics

The majority of patients in this study were comprised of hypoplastic left heart syndrome (*n* = 1089, 23%), pulmonary atresia and variants (*n* = 927, 19.6%), or complex single ventricle physiology (*n* = 855, 18.1%) (Table 1). The modified Blalock-Taussig-Thomas shunt (mBTTS) (*n* = 2224, 47%) was the most common shunt type, and the most frequently reported shunt size was 3 to <4 mm (*n* = 1657, 35%) (Table 1).

**Table 1:** Patient characteristics and outcomes.

Variable	Patients <12 mo of age *N* = 4735
Retrospective studies, *n*/39 (%)	29 (74.4)
Prospective studies, *n*/39 (%)	10 (25.6)
Patient weight (kg), median (IQR)	3.2 (3.1-3.5)
Diagnosis, *n* (%)	
Complex biventricular	214 (4.5)
Complex single ventricle	855 (18.1)
DILV/DIRV and variants	34 (0.7)
DORV and variants	75 (1.6)
d-TGA and variants	235 (5)
Heterotaxy	47 (1)
Hypoplastic left heart syndrome	1089 (23)
Hypoplastic right heart	28 (0.6)
PA and variants	927 (19.6)
TA and variants	529 (11.2)
TOF and variants	705 (14.9)
Shunt type, *n* (%)	
Blalock-Taussig-Thomas	79 (1.7)
Central	278 (5.9)
Modified Blalock-Taussig-Thomas	2224 (47)
Norwood/Modified Blalock-Taussig-Thomas	434 (9.2)
Norwood/Sano	8 (0.2)
Right ventricle-to-pulmonary artery (Sano)	48 (1)
Systemic-to-pulmonary NOS	538 (11.4)
Unknown	1126 (23.8)
Shunt size (mm), *n* (%)	
<3	4 (0.1)
3 to <4	1657 (35)
4 to <5	480 (10.1)
≥5	531 (11.2)
Unknown^a^	2063 (43.6)

DILV/DIRV, double inlet left ventricle/double inlet right ventricle; DORV, double outlet right ventricle; d-TGA, d-transposition of the great arteries; NOS, not otherwise specified; PA, pulmonary atresia; TA, tricuspid atresia; TOF, Tetralogy of Fallot.

a 240 patients from 2 studies were reported as having a shunt size range from 3 mm to 5.5 mm; therefore, individual shunt size data is unavailable for these patients.

Most patients received aspirin (*n* = 3582, 75.6%) or unfractionated heparin (UFH; *n* = 1452, 30.7%) (Table 2). A total of 724 (15.3%) patients received UFH in the immediate postoperative period and then changed to aspirin for chronic therapy. Of the 1452 who received UFH, 1204 (82.9%) were administered a mean weight average (overall range) dose of 9 (5-15) units/kg/h. The mean weighted average (overall range) aspirin dose was 5.1 (1.5-12.9) mg/kg/d among all patients (Table 2). Eight studies (*n* = 182 patients) reported aspirin resistance or platelet inhibition testing, occurring in the setting of early shunt thrombosis in most studies (*n* = 7).

**Table 2:** Postoperative (acute and chronic) antithrombotic agents and dosing.

Patients <12 months of age *N* = 4735	Agent, *n* (%)	Dosing^a^
No anticoagulation	624 (13.2)	–
Aspirin	3582 (75.6)	5.1 (1.5-12.9) mg/kg
Bivalirudin	5 (0.1)	0.1 mg/kg/h^a^
Cangrelor	25 (0.5)	0.27 (0.1-0.5) μg/kg/min
Clopidogrel	482 (10)	0.2 (0.2-0.21) mg/kg
Low molecular weight heparin (enoxaparin)	108 (2.2)	1.25 (1-1.5) mg/kg
Unfractionated heparin (all doses)	1452 (30.7)	11 units/kg/h^a^
5-15 units/kg/h	1204 (82.9)	9 units/kg/hr^a^
>15 units/kg/h	248 (17.1)	Unknown^b^
Combination of agents		
Unfractionated heparin followed by aspirin	943 (19.9)	–
Aspirin and clopidogrel	8 (0.2)	–
Unfractionated heparin/enoxaparin + aspirin	200 (4.2)	–
Unfractionated heparin/bivalirudin + cangrelor	25 (0.5)	–

a Mean weighted average (overall range).

b Heparin was titrated to a set activated partial thromboplastin time or anti-Xa level.

### 3.4 Clinical outcomes

Shunt thrombosis occurred in 390 (8.2%) patients (Table 3). Of the 17 studies that defined shunt thrombosis as early or late, there was variation in the definition of early (ranging from <24 hours to <30 days) and late shunt thrombosis (ranging from >24 hours to >3 months). Thirty studies reported shunt thrombosis occurred in 390 patients, and of these, shunt thrombosis was reported as early (*n* = 89, 22.8%) or late (*n* = 176, 45.1%) (Table 3). Bleeding and mortality related to shunt thrombosis were reported in 37 of the 39 studies and occurred in 270 (5.7%) patients and accounted for 102 of all 707 reported deaths (14.4%). Of the 390 patients who had shunt thrombosis, mortality occurred in 102 (26.2%) patients (Table 3).

**Table 3:** Clinical outcomes.

Variable	Total patients (*N* = 4735)
Shunt thrombosis, *n* (%)	390 (8.2)
Timing of shunt thrombosis^a^, *n*/390 (%)	
Early shunt thrombosis	89 (22.8)
Late shunt thrombosis	176 (45.1)
Unknown, *n*/390 (%)	105 (26.9)
Bleeding, *n* (%)	
Yes	270 (5.7)
Unknown	501 (10.6)
All-cause mortality, *n* (%)	
Yes	707 (14.9)
Unknown	376 (7.9)
Mortality attributed to shunt thrombosis^b^, *n*/390 (%)	
Yes	102 (26.2)
Unknown	0 (0)

a Definitions of early and late shunt thrombosis varied.

b Based on studies that specifically reported both rates of shunt thrombosis and shunt thrombosis-related death.

### 3.5 Meta-analysis findings

**Figure 2:**
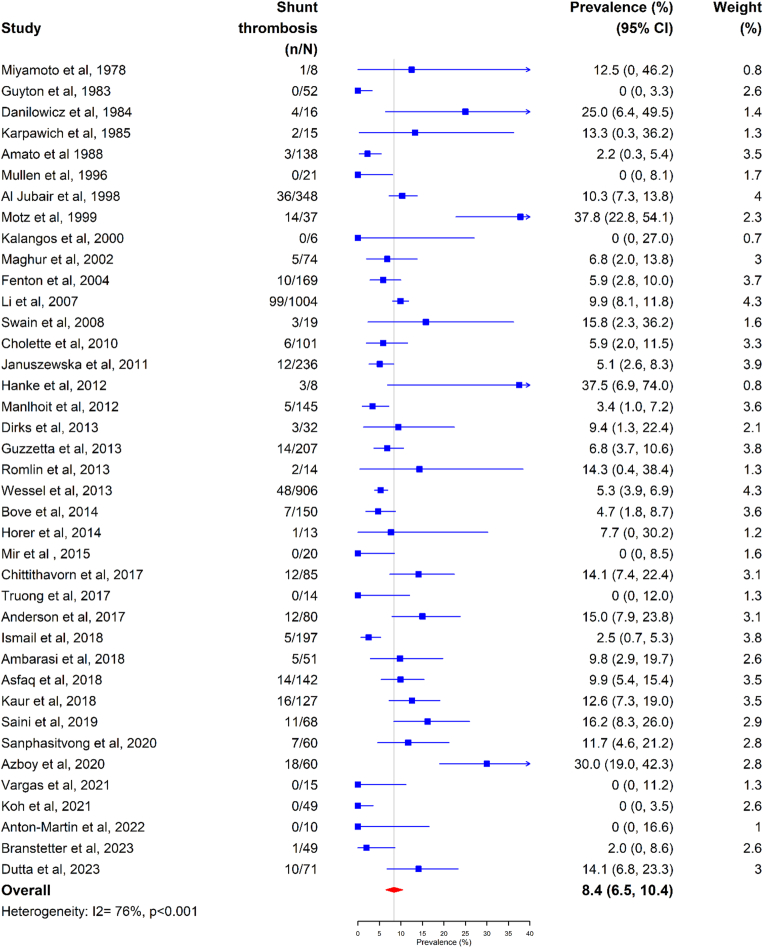
Shunt thrombosis in all studies.

The meta-analysis of the included studies demonstrated an overall pooled shunt thrombosis prevalence of 8.4% (95% CI, 6.5%-10.4%) (Figure 2). The prevalence estimate had high heterogeneity between studies (*I*^2^ = 76%, Q chi-squared test *P* < .001). When evaluating by year, studies conducted after 2009 had a lower shunt thrombosis prevalence of 7.1% (95% CI, 5.6%-10.6%) than those conducted before 2009 (8.2%; 95% CI, 5.3%-11.6%) (Supplementary Figure 1).

**Figure 3:**
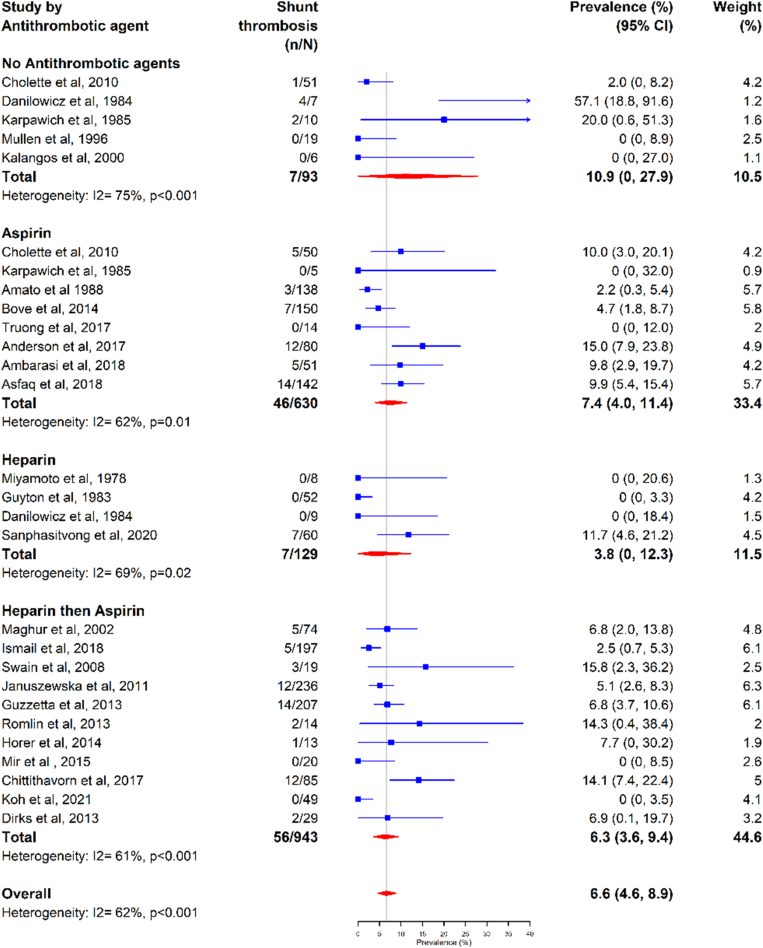
Shunt thrombosis by antithrombotic agent.

When examined by antithrombotic use, the shunt thrombosis prevalence was 10.9% (95% CI, 0%-27.9%; *I*^2^ = 75%) in patients without antithrombotic agents, 7.4% (95% CI, 4.0%-11.4%; *I*^2^ = 62%) with aspirin use, 3.8% (95% CI, 0%-12.3%; *I*^2^ = 69%) with UFH use, and 6.3% (95% CI, 3.6%-9.4%; *I*^2^ = 61%) with UFH followed by aspirin use (Figure 3).

**Figure 4:**
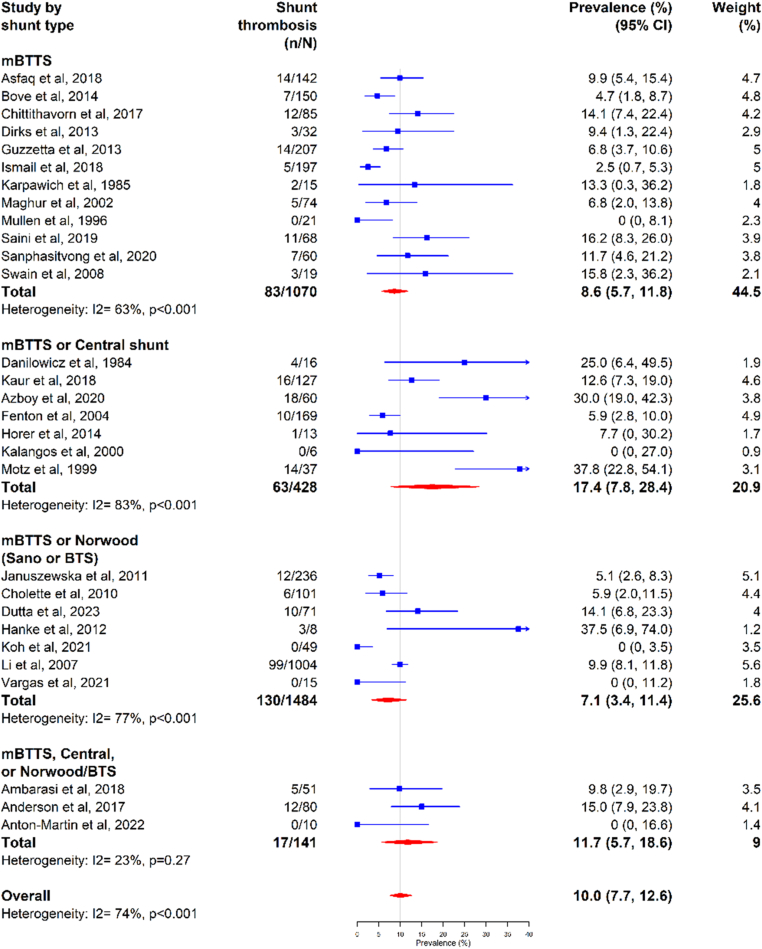
Shunt thrombosis by shunt type. BTS, Blalock Taussig Shunt; mBTTS, modified Blalock-Taussig-Thomas shunt.

Studies with central or both central and mBTTS had the highest pooled prevalence of shunt thrombosis at 17.4% (95% CI, 7.8%-28.4%; *I*^2^ = 83%), followed by those with only mBTTS at 8.6% (95% CI, 5.7%-11.8%; *I*^2^ = 63%). Studies with both mBTTS and Norwood (BTTS or Sano) had the lowest prevalence of shunt thrombosis at 7.1% (95% CI, 3.4%-11.4%; *I*^2^ = 77%) (Figure 4).

**Figure 5:**
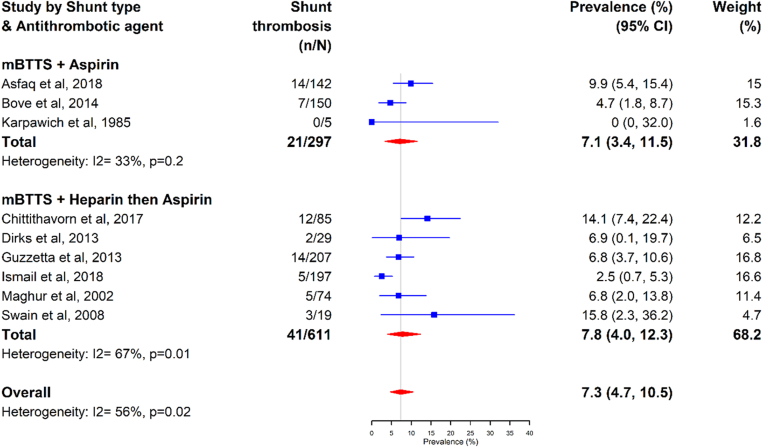
Shunt thrombosis by shunt type and antithrombic therapy. mBTTS, modified Blalock-Taussig-Thomas shunt.

When examining the prevalence of shunt thrombosis and antithrombotic therapy, patients with a mBTTS with aspirin had a shunt thrombosis prevalence of 7.1% (95% CI, 3%-11.5%; *I*^2^ = 33%), compared with those who received UFH followed by aspirin 7.8% (95% CI, 4%-12.3%; *I*^2^ = 67%) (Figure 5).

## 4 Discussion

In this systematic review investigating the type of antithrombotic strategy and shunt thrombosis, we identified a pooled prevalence of 8.4% (95% CI, 6.5%-10.4%) shunt thrombosis and a mortality rate (26.2%) in infants who developed this complication. Despite wide variability in antithrombotic regimes, aspirin was the most frequently utilized agent, alone or in combination with other agents. However, regardless of the antithrombotic regimen, patients still developed shunt thrombosis with a pooled prevalence ranging from 3.8% to 7.4%, indicating that optimal thromboprophylaxis regimens (ie, agent[s], duration, dose) to prevent this complication are still lacking.

As highlighted by this review, shunt thrombosis rates varied from 0% to 38% and appeared to be higher among patients without any antithrombotic therapy (10.9%) or those studies that included a mBTTS/central shunt (17.4%), which is similar to the results of other recent studies [4,6]. To assess potential changes in clinical practice and outcomes, we evaluated studies conducted before and after 2009 and report a slightly lower prevalence of shunt thrombosis (7.1%) than that in studies conducted before 2009 (8.9%) [50]. Of note, approximately half of the studies included definitions for early or late thrombosis, and there was a lack of standardization in how early and late shunt thrombosis was defined (eg, >24 hours, >5 days, >7 days, <30 days for early thrombosis) [2,7,12,18,20,22,[24], [26],^29^,^30^,^34^,^36^,^38^,^41^,^42^]. Therefore, any conclusions regarding shunt thrombosis should take the variability in definitions into account and is a significant limitation in the reporting of current pediatric studies. Of patients who developed shunt thrombosis, mortality was 26.2%, indicating the high potential of life-threatening complications and further associated impact of potential retrieval strategies such as extracorporeal membrane oxygenation and/or emergent cardiac catheterization.

We identified significant variability in the use of antithrombotic therapy in neonates and infants with systemic-to-pulmonary artery shunts, particularly in the early postoperative phase. Currently, various agents (eg, UFH, aspirin) are used for initial treatment, with aspirin being selected most commonly for chronic thromboprophylaxis in studies in this review [2,[17], [18], [19], [20], [21], [22], [23], [24], [25], [26], [27], [28], [29], [30], [31], [32], [33], [34], [35], [36], [37], [38], [39], [40], [41], [42], [43], [44]]. There is also variability in the dosing utilized and time to initiation. Recent studies report using UFH at a dose of 5 to 15 units/kg/h followed by the initiation of aspirin [14,21,22,24,26,[28], [29], [30],^34^,^37^,^41^,^44^]. The variability in medication and dosing is similar to that reported by other reviews evaluating antithrombic therapy in cardiac shunts [[51], [52], [53]]. A few articles note the use of intravenous antiplatelet agents in the immediate postoperative period, although further data on dosing, safety, and outcomes are urgently needed at this time [[45], [46], [47], [48], [49],^53^]. Furthermore, despite the widespread use of other antithrombotic agents in other cardiac populations, studies investigating alternative antithrombotic regimens are lacking. Understanding the different types of shunts, antithrombotic strategies, and the association between dosing strategy and thrombotic events is a priority for future research and is imperative for developing recommendations for antithrombotic therapy management to prevent thrombosis [[46], [47], [48], [49],^54^].

We report an overall 5.7% bleeding rate in the included studies. However, none of the studies reported bleeding using standard definitions. Data on bleeding according to defined criteria in the single ventricle population are lacking and limited to older single ventricle patients [[55], [56], [57], [58]]. Therefore, future efforts should evaluate not only optimal antithrombotic strategies but also use standardized definitions for reporting bleeding and thrombotic complications in this patient population.

Limitations of this systematic review include heterogeneity in the study design and patient populations of the studies included in this review, as well as variability in the definition of early and late shunt thrombosis and the definition of bleeding. Additionally, although the Sano procedure connects the right ventricle to the pulmonary artery via a small conduit, it was included in this analysis due to several studies including both BTTS/mBTTS and Sano shunts. We were unable to assess the time to initiation of aspirin due to a lack of discrete data in the included studies. Furthermore, given the paucity of patient-level (as opposed to aggregate) data and the limited number of studies reporting dosing strategy details on fixed versus titrated and dose intensity, it was not possible to investigate a potential association between these factors and incident thrombosis. We were unable to assess polycythemia, thrombocytosis, or transfusion requirements and establish any correlation with shunt thrombosis. Despite these limitations, our study followed a rigorous systematic approach to identify studies of interest and extract meaningful data to determine antithrombotic practices and outcomes in systemic-to-pulmonary artery shunts.

## 5 Conclusions

Shunt thrombosis is a recognized serious complication in neonates and infants with CHD who require a systemic-to-pulmonary artery shunt. There is an urgent need for collaborative efforts to conduct large multicenter prospective studies to evaluate optimum antithrombotic strategies for neonates and infants with systemic-to-pulmonary artery shunts and to prevent early and late shunt thrombosis.

## Appendix

**Cardiac Disease Thrombosis and Hemostasis Working Party of the ISTH SSC Subcommittee on Pediatric and Neonatal Thrombosis and Hemostasis Members:** Dalia Bashir, Kisha Beg, Victor Benvenuto, Marisol Betensky, Rukhmi Bhat, Joshua Branstetter, Paul Davies, Nhue Do, Kate Garland, Therese Giglia, Neil Goldenberg, Amy Kiskaddon, Joel Livingston, Christoph Male, Paul Monagle, Michael Silvey, Arabela Stock, Jun Teruya, Gary Woods.
