# Supplementary material for: Antithrombotic strategies and outcomes in neonates and infants with cardiac shunts: a systematic review and meta-analysis
Source: Res Pract Thromb Haemost. 2025 Aug 26;9(6):103161. doi: 10.1016/j.rpth.2025.103161 (PMC12494822; doi:10.1016/j.rpth.2025.103161)
Supplement: Supplementary Material [file mmc1.docx]

**Supplemental Appendix**

**Appendix 1: Search Strategy**

Date all searches were preformed - 12/18/2023

Date all searches were updated – 07/31/2024 (Ovid MEDLINE last upload 07/30/2024)

**Ovid MEDLINE(R) Epub Ahead of Print and In-Process, In-Data-Review & Other Non-Indexed Citations**

1 ((antiplatelet* or platelet inhibitor*or platelet aggregation Inhibitor* or thrombin inhibitor* or factor Xa or anticoagul* or antithromb* or Fibrinolytic Agent* or blood thinner* or Abciximab or Argatroban or Apixaban or Aspirin or Bivalirudin or Cangrelor or Clopidogrel or Dabigatran or Dipyridamole or Edoxaban or Enoxaparin or Eptifibatide or Heparin or Prasugrel or Rivaroxaban or Ticagrelor or Tirofiban) and (shunt* or conduit* or Blalock* or deLeval or Starnes or Melbourne or Sano or Potts* or Waterston* or Mee shunt* or BT shunt*) and (neonat* or baby or babies or infant* or newborn* or new-born* or child* or pediatric* or paediatric*)).mp.

2 exp Fibrinolytic Agents/

3 exp Anticoagulants/

4 exp Platelet Aggregation Inhibitors/

5 exp Heart Defects, Congenital/su [Surgery]

6 arteriovenous shunt, surgical/ or blalock-taussig procedure/

7 Pulmonary Artery/su [Surgery]

8 exp Aorta/su [Surgery]

9 2 or 3 or 4

10 5 or 6 or 7 or 8

11 9 and 10

12 limit 11 to "all infant (birth to 23 months)"

13 1 or 12

14 limit 13 to (english or french)

15 13 not 14

16 limit 15 to abstracts

17 14 or 16

18 remove duplicates from 17

19 limit 18 to comment

20 18 not 19

**Embase (Elsevier)**

1 'anticoagulant agent'/exp

2 'fibrinolytic agent'/exp

3 #1 OR #2

4 'systemic pulmonary shunt'/exp

5 #3 AND #4

6 #5 AND ([infant]/lim OR [newborn]/lim)

7 antiplatelet*:ti,ab,kw OR 'platelet inhibitor*or platelet aggregation inhibitor*':ti,ab,kw OR 'thrombin inhibitor*':ti,ab,kw OR 'factor xa':ti,ab,kw OR anticoagul*:ti,ab,kw OR antithromb*:ti,ab,kw OR 'fibrinolytic agent*':ti,ab,kw OR 'blood thinner*':ti,ab,kw OR abciximab:ti,ab,kw OR argatroban:ti,ab,kw OR apixaban:ti,ab,kw OR aspirin:ti,ab,kw OR bivalirudin:ti,ab,kw OR cangrelor:ti,ab,kw OR clopidogrel:ti,ab,kw OR dabigatran:ti,ab,kw OR dipyridamole:ti,ab,kw OR edoxaban:ti,ab,kw OR enoxaparin:ti,ab,kw OR eptifibatide:ti,ab,kw OR heparin:ti,ab,kw OR prasugrel:ti,ab,kw OR rivaroxaban:ti,ab,kw OR ticagrelor:ti,ab,kw OR tirofiban:ti,ab,kw

8 shunt*:ti,ab,kw OR conduit*:ti,ab,kw OR blalock*:ti,ab,kw OR delaval:ti,ab,kw OR starnes:ti,ab,kw OR melbourne:ti,ab,kw OR sano:ti,ab,kw OR potts*:ti,ab,kw OR waterston*:ti,ab,kw OR 'mee shunt*':ti,ab,kw OR 'bt shunt*':ti,ab,kw

9 neonat*:ti,ab,kw OR baby:ti,ab,kw OR babies:ti,ab,kw OR infant*:ti,ab,kw OR newborn*:ti,ab,kw OR 'new born*':ti,ab,kw OR child*:ti,ab,kw OR pediatric*:ti,ab,kw OR paediatric*:ti,ab,kw

10 #7 AND #8 AND #9

11 #6 OR #10

12 (#6 OR #10) AND ([english]/lim OR [french]/lim)

13 #11 NOT #12

14 #11 NOT #12 AND [abstracts]/lim

15 #12 OR #14

16 #15 AND ('article'/it OR 'article in press'/it OR 'review'/it OR 'short survey'/it)

**Cochrane Central Register of Controlled Trials (CENTRAL) - Wiley**

#1 MeSH descriptor: [Fibrinolytic Agents] explode all trees

#2 MeSH descriptor: [Anticoagulants] explode all trees

#3 MeSH descriptor: [Platelet Aggregation Inhibitors] explode all trees

#4 MeSH descriptor: [Heart Defects, Congenital] explode all trees and with qualifier(s): [surgery - SU]

#5 MeSH descriptor: [Blalock-Taussig Procedure] this term only

#6 MeSH descriptor: [Arteriovenous Shunt, Surgical] this term only

#7 MeSH descriptor: [Pulmonary Artery] explode all trees and with qualifier(s): [surgery - SU]

#8 MeSH descriptor: [Aorta] explode all trees and with qualifier(s): [surgery - SU]

#9 MeSH descriptor: [Infant] explode all trees

#10 (neonat* or baby or babies or infant* or newborn* or new-born* or child* or pediatric* or paediatric*):ti,ab,kw

#11 (antiplatelet* or platelet inhibitor*or platelet aggregation Inhibitor* or thrombin inhibitor* or factor Xa or anticoagul* or antithromb* or fibrinolytic agent* or blood thinner* or Abciximab or Argatroban or Apixaban or Aspirin or Bivalirudin or Cangrelor or Clopidogrel or Dabigatran or Dipyridamole or Edoxaban or Enoxaparin or Eptifibatide or Heparin or Prasugrel or Rivaroxaban or Ticagrelor or Tirofiban):ti,ab,kw

#12 (shunt* or conduit* or Blalock* or deLeval or Starnes or Melbourne or Sano or Potts* or Waterston* or Mee shunt* or BT shunt*):ti,ab,kw

#13 #1 OR #2 OR #3

#14 #4 or #5 or #6 or #7 or #8

#15 #13 AND # 14 AND #9

#16 #10 AND #11 AND #12

#17 #15 OR #16

Supplemental Figure 1. Shunt Thrombosis Before and After 2009


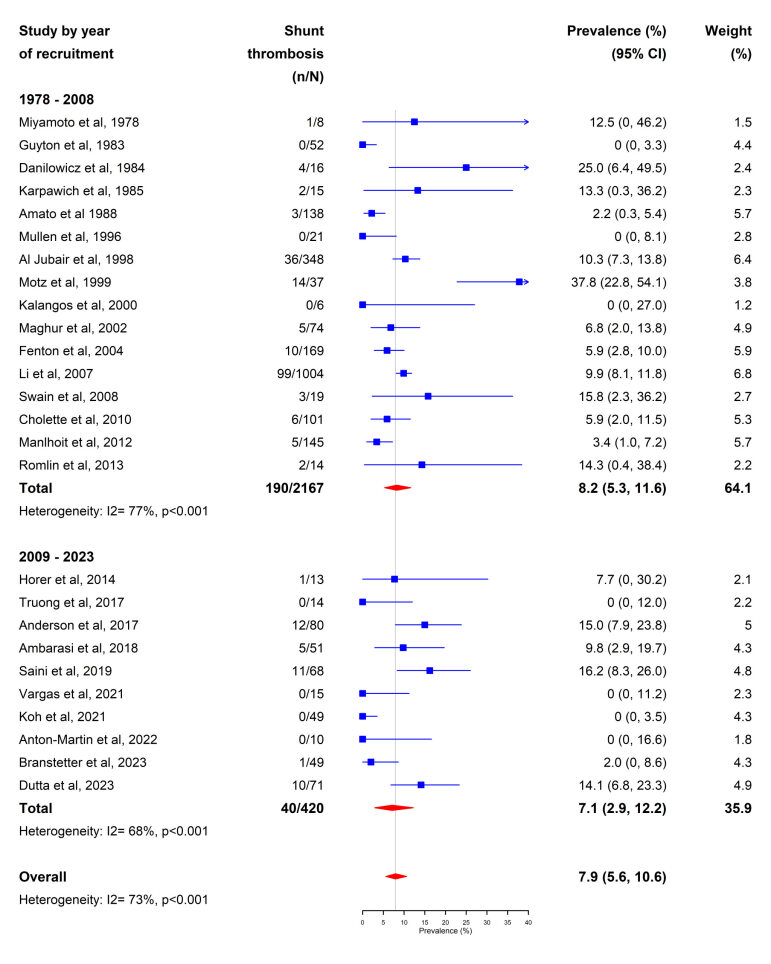


**Supplemental Table 1: Studies Included**

|  | **Quality** | **Study Design** | | **N** | **Age** | | | **Weight** | | **Congenital Heart Disease Diagnosis** | | | | | | | | | | |
| --- | --- | --- | --- | --- | --- | --- | --- | --- | --- | --- | --- | --- | --- | --- | --- | --- | --- | --- | --- | --- |
| **Authors** |  | **Design** | **Type** | **Total** | **Neonate (n)** | **Infant (n)** | **Un-known (n)** | **kg** | **Un-known (n)** | **TOF (n)** | **PA (n)** | **TA (n)** | **DORV (n)** | **DIVL/DIRV (n)** | **Hetero-taxy (n)** | **dTGA (n)** | **HLHS (n)** | **HR (n)** | **Complex SV (n)** | **Complex BV (n)** |
| Miyamoto et al, 1978 | Poor | retrospective | cohort | 8 | 5 | 3 | 0 | 4.3 | 0 | 2 | 3 | 0 | 0 | 0 | 0 | 3 | 0 | 0 | 0 | 0 |
| Guyton et al, 1983 | Fair | retrospective | cohort | 53 | 24 | 29 | 0 | - | 53 | 19 | 9 | 4 | 0 | 0 | 0 | 3 | 0 | 0 | 19 | 0 |
| Danilowicz et al, 1984 | Poor | retrospective | cohort | 16 | 16 | 0 | 0 | 2.9 | 0 | 1 | 11 | 2 | 0 | 0 | 0 | 0 | 0 | 0 | 2 | 0 |
| Karpawich et al, 1985 | Fair | prospective | cohort | 15 | 7 | 8 | 0 | - | 15 | 8 | 3 | 1 | 1 | 0 | 0 | 0 | 0 | 0 | 2 | 0 |
| Amato et al 1988 | Fair | retrospective | cohort | 138 | 75 | 13 | 50 | - | 138 | 11 | 41 | 12 | 1 | 0 | 0 | 3 | 1 | 0 | 6 | 1 |
| Mullen et al, 1996 | Fair | retrospective | cohort | 21 | 6 | 15 | 0 | 5.1 | 0 | 0 | 0 | 0 | 0 | 0 | 0 | 0 | 0 | 0 | 0 | 0 |
| Al Jubair et al, 1998 | Good | retrospective | cohort | 348 | 78 | 0 | 270 | - | 348 | 114 | 221 | 64 | 0 | 0 | 0 | 171 | 0 | 0 | 268 | 0 |
| Motz et al, 1999 | Fair | retrospective/prospective | cohort | 37 | 0 | 37 | 0 | 4.3 | 0 | 0 | 0 | 0 | 0 | 0 | 0 | 0 | 0 | 0 | 0 | 0 |
| Kalangos et al, 2000 | Poor | retrospective | cohort | 6 | 6 | 0 | 0 | 2.7 | 0 | 2 | 2 | 0 | 0 | 0 | 0 | 0 | 0 | 0 | 2 | 0 |
| Maghur et al, 2002 | Fair | retrospective | cohort | 74 | 25 | 49 | 0 | - | 74 | 25 | 9 | 31 | 0 | 0 | 0 | 0 | 0 | 0 | 9 | 0 |
| Fenton et al, 2004 | Good | retrospective | cohort | 169 | 169 | 0 | 0 | 3.2 | 0 | 0 | 36 | 0 | 0 | 0 | 0 | 0 | 52 | 0 | 23 | 58 |
| Li et al, 2007 | Good | prospective | cohort | 1004 | 1004 | 0 | 0 | - | 1004 | 127 | 177 | 103 | 0 | 0 | 38 | 0 | 346 | 0 | 213 | 0 |
| Swain et al, 2008 | Fair | retrospective | cohort | 19 | 19 | 0 | 0 | 3.24 | 0 | 11 | 2 | 4 | 1 | 0 | 0 | 1 | 0 | 0 | 0 | 0 |
| Cholette et al, 2010 | Good | prospective | cohort | 19 | 5 | 14 | 0 | 3.3 | 0 | 1 | 1 | 0 | 0 | 0 | 0 | 1 | 2 | 1 | 13 | 0 |
| Januszewska et al, 2011 | Poor | retrospective | cohort | 236 | 236 | 0 | 0 | 3.4 | 0 | 0 | 0 | 0 | 0 | 0 | 0 | 0 | 236 | 0 | 0 | 0 |
| Hanke et al, 2012 | Good | prospective | cohort | 7 | 2 | 5 | 0 | - | 7 | 0 | 0 | 1 | 1 | 1 | 0 | 0 | 4 | 0 | 0 | 0 |
| Manlhoit et al, 2012 | Good | retrospective | cohort | 145 | 145 | 0 | 0 | - | 145 | 0 | 0 | 27 | 2 | 13 | 0 | 0 | 101 | 0 | 2 | 0 |
| Dirks et al, 2013 | Fair | retrospective | cohort | 32 | 26 | 6 | 0 | 2.9 | 0 | 0 | 0 | 0 | 0 | 0 | 0 | 0 | 0 | 0 | 11 | 21 |
| Guzzetta et al, 2013 | Good | retrospective | cohort | 207 | 162 | 45 | 0 | 3.1 | 0 | 0 | 0 | 0 | 0 | 0 | 0 | 0 | 0 | 0 | 0 | 0 |
| Romlin et al, 2013 | Fair | prospective | cohort | 14 | 12 | 2 | 0 | 3.5 | 0 | 0 | 4 | 0 | 1 | 0 | 0 | 0 | 7 | 0 | 0 | 2 |
| Wessel et al, 2013 | Good | prospective | randomized | 906 | 0 | 0 | 906 | 3.4 | 0 | 198 | 152 | 105 | 0 | 0 | 0 | 0 | 224 | 0 | 151 | 38 |
| Bove et al, 2014 | Fair | retrospective | cohort | 150 | 150 | 0 | 0 | 3.1 | 0 | 44 | 45 | 0 | 0 | 0 | 0 | 12 | 0 | 0 | 47 | 2 |
| Horer et al, 2014 | Fair | retrospective | cohort | 13 | 13 | 0 | 0 | 0 | 13 | 1 | 6 | 2 | 1 | 0 | 0 | 1 | 1 | 0 | 1 | 0 |
| Mir et al , 2015 | Fair | prospective | cohort | 20 | 20 | 0 | 0 | 3.08 | 0 | 0 | 1 | 2 | 0 | 3 | 3 | 0 | 11 | 0 | 0 | 0 |
| Chittithavorn et al, 2017 | Good | retrospective | cohort | 85 | 85 | 0 | 0 | 2.8 | 0 | 0 | 41 | 13 | 0 | 0 | 0 | 0 | 0 | 0 | 27 | 4 |
| Truong et al, 2017 | Fair | prospective | cohort | 14 | 0 | 0 | 14 | 4 | 0 | 2 | 4 | 4 | 0 | 2 | 0 | 2 | 9 | 0 | 1 | 0 |
| Anderson et al, 2017 | Good | retrospective | cohort | 80 | 0 | 0 | 80 | 0 | 80 | 0 | 27 | 11 | 0 | 0 | 0 | 0 | 16 | 0 | 14 | 12 |
| Ismail et al, 2018 | Fair | retrospective | cohort | 197 | 119 | 78 | 0 | 4.6 | 0 | 9 | 0 | 104 | 26 | 12 | 0 | 28 | 0 | 0 | 5 | 12 |
| Ambarasi et al, 2018 | Fair | retrospective | cohort | 51 | 0 | 0 | 51 | 3.2 | 0 | 6 | 5 | 0 | 0 | 0 | 6 | 3 | 12 | 19 | 0 | 0 |
| Asfaq et al, 2018 | Fair | retrospective | cohort | 142 | 0 | 0 | 142 | - | 142 | 45 | 39 | 9 | 3 | 0 | 0 | 2 | 10 | 0 | 0 | 34 |
| Kaur et al, 2018 | Fair | retrospective | cohort | 127 | 0 | 0 | 127 | - | 127 | 30 | 20 | 22 | 22 | 0 | 0 | 0 | 0 | 0 | 0 | 15 |
| Saini et al, 2019 | Good | retrospective | cohort | 68 | 68 | 0 | 0 | 3.35 | 0 | 21 | 14 | 11 | 5 | 0 | 0 | 0 | 8 | 0 | 5 | 4 |
| Sanphasitvong et al, 2020 | Fair | retrospective | cohort | 60 | 60 | 0 | 0 | 3.2 | 60 | 11 | 19 | 0 | 4 | 0 | 0 | 3 | 0 | 0 | 23 | 0 |
| Azboy et al, 2020 | Good | retrospective | cohort | 60 | 28 | 32 | 0 | 3.3 | 0 | 10 | 31 | 0 | 5 | 0 | 0 | 7 | 0 | 0 | 0 | 7 |
| Vargas et al, 2021 | Good | prospective | cohort | 15 | 15 | 0 | 0 | 3.0 | 0.0 | 1 | 1 | 0 | 0 | 0 | 0 | 0 | 0 | 7 | 5 | 1 |
| Koh et al, 2021 | Fair | retrospective | cohort | 49 | 41 | 8 | 0 | 2.8 | 0 | 6 | 1 | 4 | 7 | 1 | 0 | 3 | 21 | 1 | 3 | 2 |
| Anton-Martin et al, 2022 | Fair | retrospective | cohort | 10 | 10 | 0 | 0 | 3.49 | 0 | 0 | 3 | 0 | 0 | 0 | 0 | 0 | 6 | 0 | 1 | 0 |
| Branstetter et al, 2023 | Fair | retrospective | cohort | 49 | 49 | 0 | 0 | 3.1 | 0 | 0 | 3 | 0 | 0 | 2 | 0 | 0 | 29 | 0 | 7 | 8 |
| Dutta et al, 2023 | Good | retrospective | cohort | 71 | 0 | 0 | 71 | - | 71 | 0 | 0 | 0 | 0 | 0 | 0 | 0 | 0 | 0 | 0 | 0 |

|  | **Shunt Type** | | | | | | | **Shunt Size** | | | | **Antithrombotic Strategy** | | | | | | | | | | |
| --- | --- | --- | --- | --- | --- | --- | --- | --- | --- | --- | --- | --- | --- | --- | --- | --- | --- | --- | --- | --- | --- | --- |
|  | BTT | mBTT | Central | RV-PA | Norwood/  mBTT | Norwood/ Sanno | Un-known | <3 | 3 to <4 | 4 to <5 | ≥5 | None (n) | ASA (n) | ASA dose (mg/kg) | UFH (n) | UFH (u/kg/hr) | Bivalirudin (n) | Bivalirudin (mg/kg/hr) | Enoxaparin (n) | Enoxaparin (mg/kg) | Clopidogrel (n) | Clopidogrel (mg/kg) |
| Miyamoto et al, 1978 | 0 | 0 | 0 | 0 | 0 | 0 | 8 | 0 | 0 | 1 | 7 | 1 | 0 | - | 8 | 5 | 0 | - | 0 | - | 0 | - |
| Guyton et al, 1983 | 53 | 0 | 0 | 0 | 0 | 0 | 0 | 0 | 0 | 0 | 0 | 12 | 0 | - | 41 | 25 | 0 | - | 0 | - | 0 | - |
| Danilowicz et al, 1984 | 0 | 0 | 16 | 0 | 0 | 0 | 0 | 1 | 2 | 7 | 6 | 7 | 0 | - | 9 | - | 0 | - | 0 | - | 0 | - |
| Karpawich et al, 1985 | 0 | 15 | 0 | 0 | 0 | 0 | 0 | 0 | 0 | 8 | 7 | 10 | 5 | 10 | 0 | - | 0 | - | 0 | - | 0 | - |
| Amato et al 1988 | 26 | 69 | 61 | 0 | 0 | 0 | 0 | 0 | 0 | 0 | 0 | - | 138 | 10 | 0 | - | 0 | - | 0 | - | 0 | - |
| Mullen et al, 1996 | 0 | 21 | 0 | 0 | 0 | 0 | 0 | 0 | 0 | 0 | 23 | 19 | 2 | 5 | 0 | - | 0 | - | 0 | - | 0 | - |
| Al Jubair et al, 1998 | 0 | 0 | 0 | 0 | 0 | 0 | 0 | 0 | 0 | 85 | 123 | 208 | 150 | 1.5 | 221 | 15 | 0 | - | 0 | - | 0 | - |
| Motz et al, 1999 | 0 | 9 | 28 | 0 | 0 | 0 | 0 | 0 | 0 | 28 | 9 | 3 | 34 | 2 | 0 | - | 0 | - | 0 | - | 0 | - |
| Kalangos et al, 2000 | 0 | 3 | 3 | 0 | 0 | 0 | 0 | 0 | 3 | 3 | 0 | 6 | 0 | - | 0 | - | 0 | - | 0 | - | 0 | - |
| Maghur et al, 2002 | 0 | 74 | 0 | 0 | 0 | 0 | 0 | 0 | 0 | 32 | 42 | - | 74 | 5 | 74 | 8 | 0 | - | 0 | - | 0 | - |
| Fenton et al, 2004 | 0 | 168 | 1 | 0 | 0 | 0 | 0 | 0 | 3-5 | 0 | 0 | 115 | 54 | 5 | 0 | - | 0 | - | 0 | - | 0 | - |
| Li et al, 2007 | 0 | 0 | 0 | 0 | 323 | 50 | 0 | 0 | 1004 | 0 | 0 | 198 | 806 | - | 0 | - | 0 | - | 0 | - | 0 | - |
| Swain et al, 2008 | 0 | 19 | 0 | 0 | 0 | 0 | 0 | 0 | 1 | 18 | 0 | - | 19 | 5 | 19 | 10 | 0 | - | 0 | - | 0 | - |
| Cholette et al, 2010 | 0 | 3 | 0 | 0 | 1 | 1 | 0 | 0 | 0 | 0 | 0 | 1 | 5 | 10 | 0 | - | 0 | - | 0 | - | 0 | - |
| Januszewska et al, 2011 | 0 | 0 | 0 | 0 | 0 | 236 | 0 | 0 | 0 | 3 | 233 | - | 236 | 3.5 | 236 | 7.5 | 0 | - | 0 | - | 0 | - |
| Hanke et al, 2012 | 0 | 2 | 0 | 0 | 2 | 1 | 0 | 0 | 0 | 0 | 0 | - | 8.0 | 3.2 | 0 | - | 0 | - | 0 |  | 8 | 0.206 |
| Manlhoit et al, 2012 | 0 | 80 | 0 | 16 | 0 | 0 | 0 | 0 | 0 | 0 | 0 | 44 | 3 | 5 | 0 | - | 0 | - | 95 | 1.25 | 0 | - |
| Dirks et al, 2013 | 0 | 32 | 0 | 0 | 0 | 0 | 0 | 0 | 27 | 5 | 0 | - | 22 | 4 | 30 | 7.5 | 0 | - | 7 | 1.25 | 0 | - |
| Guzzetta et al, 2013 | 0 | 207 | 0 | 0 | 0 | 0 | 0 | 0 | 198 | 8 | 1 | - | 207 | 13 | 207 | 10 | 0 | - |  | - | 0 | - |
| Romlin et al, 2013 | 0 | 4 | 1 | 2 | 1 | 6 | 0 | 0 | 0 | 0 | 0 | - | 14 | 4.9 | 14 | 11 | 0 | - |  | - | 0 | - |
| Wessel et al, 2013 | 0 | 509 | 78 | 7 | 113 | 114 | 0 | 0 | 0 | 0 | 0 | - | 760 | 4 | 0 | - | 0 | - |  | - | 467 | 0.2 |
| Bove et al, 2014 | 0 | 150 | 0 | 0 | 0 | 0 | 0 | 0 | 16 | 65 | 63 | - | 150 | 5 | 0 | - | 0 | - |  | - | 0 | - |
| Horer et al, 2014 | 0 | 7 | 6 | 0 | 0 | 0 | 0 | 0 | 13 | 0 | 0 | - | 13 | 4 | 13 | - | 0 | - |  | - | 0 | - |
| Mir et al , 2015 | 0 | 6 | 0 | 5 | 9 | 0 | 0 | 0 | 0 | 0 | 0 | - | 20 | 6 | 20 | - | 0 | - |  | - | 0 | - |
| Chittithavorn et al, 2017 | 0 | 85 | 0 | 0 | 0 | 0 | 0 | 0 | 62 | 23 | 0 | - | 85 | 5 | 85 | 10 | 0 | - |  | - | 0 | - |
| Truong et al, 2017 | 0 | 6 | 2 | 0 | 1 | 5 | 0 | 0 | 0 | 0 | 0 | - | 14 | 4 | 0 | - | 0 | - |  | - | 0 | - |
| Anderson et al, 2017 | 0 | 39 | 12 | 0 | 28 | 0 | 0 | 0 | 46 | 27 | 5 | - | 80 | 7 | 0 | - | 0 | - |  | - | 0 | - |
| Ismail et al, 2018 | 0 | 197 | 0 | 0 | 0 | 0 | 0 | 0 | 0 | 0 | 0 | - | 197 | 4 | 197 | 15 | 0 | - |  | - | 0 | - |
| Ambarasi et al, 2018 | 0 | 4 | 35 | 0 | 12 | 0 | 0 | 0 | 51 | 0 | 0 | - | 51 | 3 | 0 | - | 0 | - |  | - | 0 | - |
| Asfaq et al, 2018 | 0 | 142 | 0 | 0 | 0 | 0 | 0 | 0 | 67 | 75 | 0 | - | 142 | 5 | 0 | - | 0 | - |  | - | 0 | - |
| Kaur et al, 2018 | 0 | 106 | 21 | 0 | 0 | 0 | 0 | 3 | 79 | 42 | 3 | - | 127 | 2 | 0 | - | 0 | - |  | - | 0 | - |
| Saini et al, 2019 | 0 | 68 | 0 | 0 | 0 | 0 | 0 | 0 | 37 | 31 | 0 | - | 68 | 7.5 | 64 | 10 | 0 | - | 68 | 1.25 | 0 | - |
| Sanphasitvong et al, 2020 | 0 | 60 | 0 | 0 | 0 | 0 | 0 | 0 | 46 | 14 | 0 | - | 0 | - | 60 | 19 | 0 | - | 0 | - | 0 | - |
| Azboy et al, 2020 | 0 | 47 | 13 | 0 | 0 | 0 | 0 | 0 | 0 | 0 | 0 | - | 0 | - | 0 | - | 0 | - | 0 | - | 0 | - |
| Vargas et al, 2021 | 0 | 3 | 0 | 0 | 5 | 6 | 0 | 0 | 0 | 0 | 0 | - | 0 | - | 0 | - | 0 | - | 0 | - | 0 | - |
| Koh et al, 2021 | 0 | 13 | 0 | 0 | 23 | 6 | 0 | 0 | 0 | 0 | 0 | - | 49 | 8 | 49 | - | 0 | - | 0 | - | 0 | - |
| Anton-Martin et al, 2022 | 0 | 3 | 1 | 0 | 6 | 0 | 0 | 0 | 5 | 5 | 0 | - | 0 | - | 5 | 16 | 5 | 0.1 | 0 | - | 0 | - |
| Branstetter et al, 2023 | 0 | 30 | 0 | 18 | 0 | 0 | 0 | 0 | 0 | 0 | 0 | - | 49 | 6 | 49 | 15 | 0 | - | 0 | - | 0 | - |
| Dutta et al, 2023 | 0 | 43 | 0 | 0 | 14 | 9 | 0 | 0 | 0 | 3.5-5 | 9 | - | 0 | - | 51 | - | 0 | - | 0 | - | 0 | - |

|  | **Shunt Thrombosis** | | | | **Mortality** | | **Bleeding** | |
| --- | --- | --- | --- | --- | --- | --- | --- | --- |
| **Authors** | **All** | **Early** | **Late** | **Unknown** | **Related to shunt thrombosis** | **Unknown** |  | **Unknown** |
| Miyamoto et al, 1978 | 1 | 1 | 0 | 0 | 0 | 0 | 1 | 0 |
| Guyton et al, 1983 | 0 | 0 | 0 | 0 | 0 | 0 | 6 | 0 |
| Danilowicz et al, 1984 | 5 | 4 | 1 | 0 | 4 | 0 | 0 | 0 |
| Karpawich et al, 1985 | 2 | 0 | 2 | 0 | 0 | 0 | 0 | 0 |
| Amato et al 1988 | 3 | 1 | 2 | 0 | 1 | 0 | 0 | 0 |
| Mullen et al, 1996 | 0 | 0 | 0 | 0 | 0 | 0 | 0 | 0 |
| Al Jubair et al, 1998 | 36 | 10 | 26 | 0 | 15 | 0 | 0 | 0 |
| Motz et al, 1999 | 14 | 1 | 13 | 0 | 4 | 0 | 0 | 0 |
| Kalangos et al, 2000 | 0 | 0 | 0 | 0 | 0 | 0 | 0 | 0 |
| Maghur et al, 2002 | 5 | 3 | 2 | 0 | 4 | 0 | 8 | 0 |
| Fenton et al, 2004 | 10 | 0 | 10 | 0 | 5 | 0 | 0 | 0 |
| Li et al, 2007 | 99 | 0 | 99 | 0 | 0 | 223 | 0 | 0 |
| Swain et al, 2008 | 3 | 3 | 0 | 0 | 0 | 0 | 1 | 0 |
| Cholette et al, 2010 | 6 | 6 | 0 | 0 | 0 | 19 | 0 | 0 |
| Januszewska et al, 2011 | 12 | 3 | 9 | 0 | 5 | 0 | 0 | 0 |
| Hanke et al, 2012 | 3 | 3 | 0 | 0 | 0 | 0 | 1 | 0 |
| Manlhoit et al, 2012 | 5 | 0 | 0 | 5 | 13 | 0 | 2 | 0 |
| Dirks et al, 2013 | 3 | 3 | 0 | 0 | 0 | 0 | 1 | 0 |
| Guzzetta et al, 2013 | 14 | 14 | 0 | 0 | 3 | 0 | 0 | 0 |
| Romlin et al, 2013 | 2 | 0 | 2 | 0 | 0 | 0 | 0 | 14 |
| Wessel et al, 2013 | 48 | 0 | 0 | 48 | 0 | 116 | 176 | 0 |
| Bove et al, 2014 | 7 | 7 | 0 | 0 | 7 | 0 | 0 | 150 |
| Horer et al, 2014 | 1 | 0 | 1 | 0 | 1 | 0 | 0 | 0 |
| Mir et al , 2015 | 0 | 0 | 0 | 20 | 0 | 0 | 0 | 0 |
| Chittithavorn et al, 2017 | 12 | 0 | 0 | 12 | 8 | 0 | 3 | 0 |
| Truong et al, 2017 | 0 | 0 | 0 | 0 | 0 | 0 | 7 | 0 |
| Anderson et al, 2017 | 12 | 0 | 0 | 12 | 2 | 0 | 0 | 0 |
| Ismail et al, 2018 | 5 | 5 | 0 | 0 | 1 | 0 | 0 | 0 |
| Ambarasi et al, 2018 | 5 | 4 | 1 | 0 | 0 | 5 | 0 | 0 |
| Asfaq et al, 2018 | 14 | 0 | 0 | 14 | 14 | 0 | 0 | 0 |
| Kaur et al, 2018 | 16 | 0 | 0 | 16 | 4 | 0 | 0 | 0 |
| Saini et al, 2019 | 11 | 0 | 0 | 11 | 0 | 7 | 5 | 0 |
| Sanphasitvong et al, 2020 | 7 | 0 | 0 | 7 | 1 | 0 | 5 | 0 |
| Azboy et al, 2020 | 18 | 10 | 8 | 0 | 5 | 0 | 3 | 0 |
| Vargas et al, 2021 | 0 | 0 | 0 | 0 | 0 | 0 | 5 | 0 |
| Koh et al, 2021 | 0 | 0 | 0 | 0 | 0 | 0 | 0 | 0 |
| Anton-Martin et al, 2022 | 0 | 0 | 0 | 0 | 0 | 0 | 1 | 0 |
| Branstetter et al, 2023 | 1 | 1 | 0 | 0 | 0 | 6 | 29 | 0 |
| Dutta et al, 2023 | 10 | 10 | 0 | 0 | 5 | 0 | 16 | 0 |
